# Carpal Tunnel Syndrome in the Setting of Ulnar Dysmelia

**DOI:** 10.1016/j.jhsg.2022.07.003

**Published:** 2022-08-13

**Authors:** Rachel J. Milam, Nicholas J. Drayer, Matthew P. Garries, Sean D. Ghidella

**Affiliations:** ∗ Department of Orthopaedics, Madigan Army Medical Center, Tacoma, WA; † Department of Orthopaedics, Winn Army Community Hospital, Fort Stewart, GA; ‡ Lake Erie College of Osteopathic Medicine, Erie, PA; § Puget Sound Orthopaedics, Tacoma, WA

**Keywords:** Carpal tunnel release, Carpal tunnel syndrome, Ulnar dysmelia, Ulnar hemimelia

## Abstract

Ulnar dysmelia is a congenital anatomic disorder characterized by abnormal development of the ulna and subsequent distal bones. This rare disorder has a heterogeneous presentation in patients described in the literature. We present the case of a 23-year-old woman with ulnar dysmelia who lacked the ulnar attachments of the transverse carpal ligament and developed carpal tunnel syndrome at a relatively young age, requiring carpal tunnel release. This case report presents an interesting and unique cause of carpal tunnel syndrome and reviews the literature on ulnar dysmelia.

Carpal tunnel syndrome (CTS) is an entrapment neuropathy of the upper extremities and can lead to considerable disability and functional impairment. It is a commonly occurring neuropathy and was found to have a prevalence of 2%–4% in 1 Swedish epidemiologic study.^1^ Its pathophysiology involves increased pressure within the carpal tunnel, leading to changes in the microcirculation and nerve demyelination. An important component of the disease process is the anatomy of the carpal tunnel, which is defined by the carpal bones dorsally and by the transverse carpal ligament (TCL) volarly. The TCL has 4 points of attachment: proximally to the scaphoid tubercle and pisiform and distally to the hook of the hamate and the trapezium. These attachments remained consistent across 5 cadaveric limbs in 1 anatomic study.^2^ The tension across the TCL restricts the volume of the carpal tunnel. When edema develops because of inflammation, the increased pressure is confined to the carpal tunnel and damages the median nerve.

Intuitively, one would infer that if these attachments were absent, the space would not be as definitively constrained and CTS might not develop. However, here, we present a case of CTS in a 23-year-old female patient with congenital ulnar dysmelia who developed CTS despite the absence of the ulna, hamate, and triquetrum. Thus, she lacked the ulnar attachments of the carpal tunnel.

## Case

A right-handed 23-year-old woman with a history of bilateral, congenital ulnar deficiency presented for evaluation with a complaint of a 1-year history of constant left wrist pain (scored as 3/10). To her knowledge, she had received no prior interventions for her congenital dysplasia. There was associated numbness and paresthesia about the palmar aspect of her left hand. Her symptoms were exacerbated by typing and sleeping but improved with shaking and repositioning. By this point, nonsurgical management had failed in the patient. On physical examination, the patient had 30°–135° of active elbow flexion. She had a 50° arc of pronation-supination centered on supination. At rest, her wrist was held in a flexed position that passively corrected to, but not past, the neutral position. She demonstrated active wrist flexion to 80° but lacked active extension past 0°. There was a 40° ulnar deviation contracture and no neurovascular deficits. The patient had positive Phalen and Durkan test results. On the right side, she had 40°–135° of elbow flexion, a similar pronation-supination arc, wrist flexion to 90°, and wrist extension to positive 30° beyond the neutral position. Her right wrist had a less severe ulnar deviation contracture of 30°.

**Figure 1:**
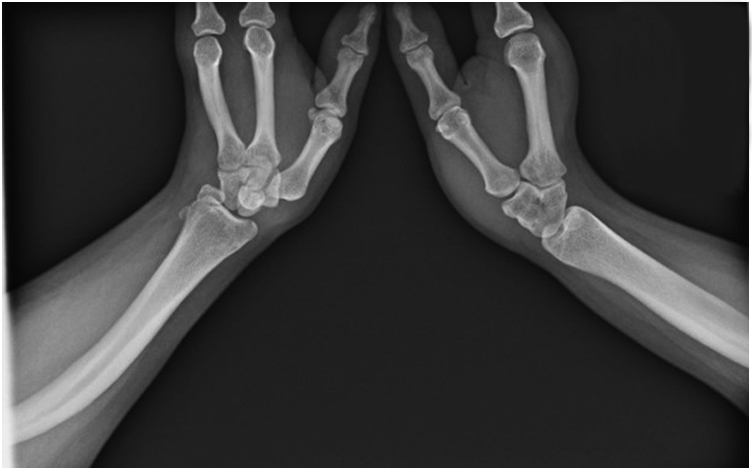
Bilateral wrist radiograph. A posteroanterior radiograph of the bilateral wrists demonstrates single bone forearms, presumed to be the radius, wrist coalitions, and the presence of rays 1–3 on the left side and rays 1–2 on the right side.

**Figure 2:**
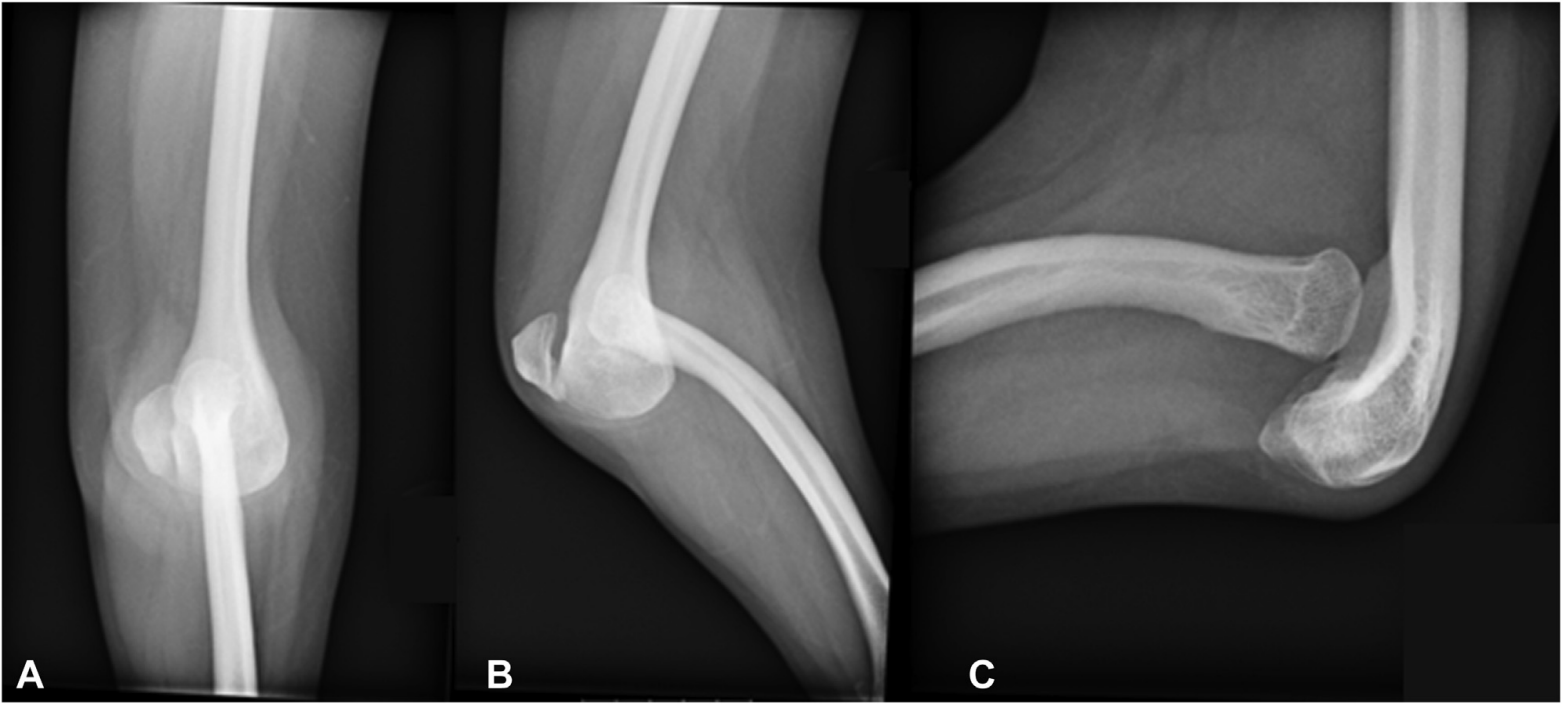
Left elbow radiographs. **A** Anteroposterior, **B** oblique, and **C** lateral radiographs of the left elbow demonstrate an abnormally developed elbow with an anteriorly dislocated, bowed radius forming a pseudoarticulation with the humerus and an abnormally rounded distal humerus. There is an ossific density near the distal humerus that is presumed to be the ulnar remnant.

Radiographic evaluation demonstrated dysplasia of the ulna bilaterally, with rays 1–3 present on the left hand and 1–2 present on the right hand, and coalition of the carpal structures (Fig. 1). The proximal radius was dislocated anteriorly and formed a pseudoarticulation with the distal humerus. The trochlea of the distal humerus was dysmorphic and rounded, and there was a small boney remnant adjacent to the trochlea, which likely represented the remnant ulna (Fig. 2).

Electrodiagnostic studies of the median nerve at the level of the wrist demonstrated a distal sensory latency of 3.9 milliseconds with an amplitude of 8.1 μV and a motor latency of 4.8 milliseconds with an amplitude of 0.8 mV, consistent with severe left median neuropathy at the carpal tunnel. Magnetic resonance imaging of the left wrist was performed to better assess the anatomy of the region of the median nerve compression. This demonstrated the congenital absence of the ulnar structures of the wrist. The flexor compartment appeared as a sac that was fully encircled by a fascial plane and a thickening, likely representing a TCL analog, despite the absence of the hamate or triquetrum.

**Figure 3:**
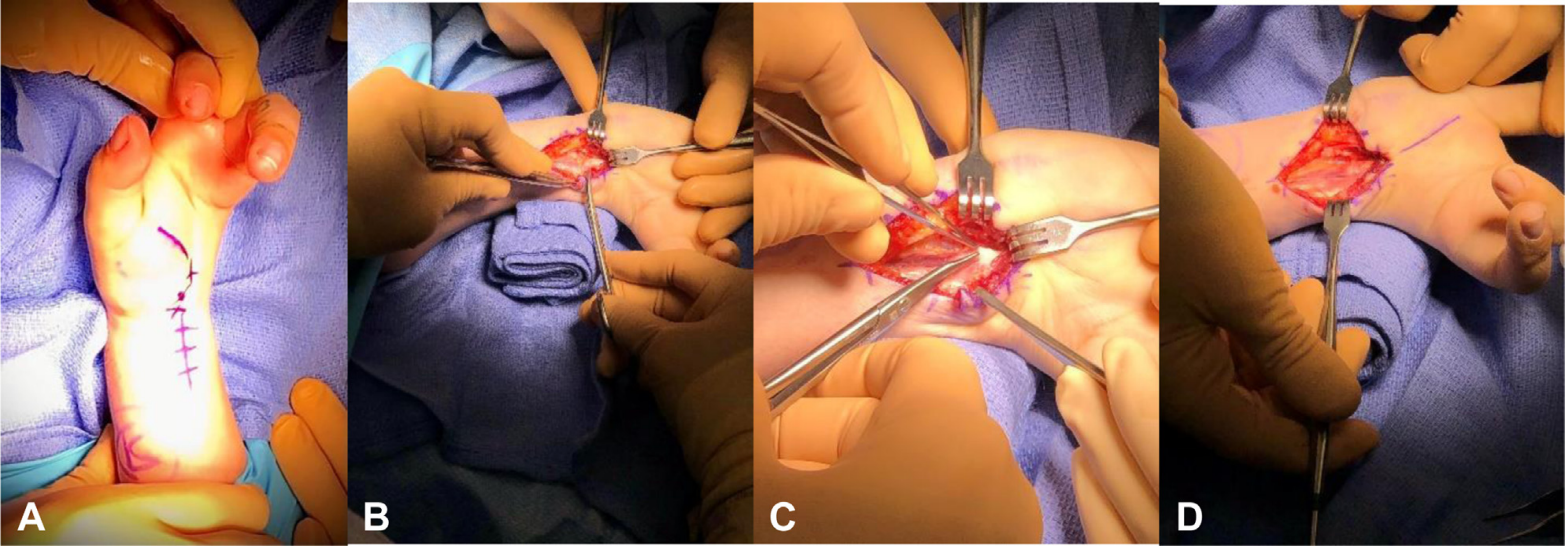
Intraoperative images. Intraoperative images taken during carpal tunnel release demonstrating **A** the planned incision, **B** dissection down to the fascial plane, **C** release of the TCL analog, and **D** contents after the release.

The constellation of symptoms, examination, and imaging was consistent with CTS on the left wrist, and the patient consented for surgical intervention. She underwent open carpal tunnel release through an extended incision to ensure adequate visualization (Fig. 3). The dissection was carried down to the fascial plane, and the fascia was released from the proximal aspect to the distal aspect until the thickening was encountered, representing the TCL analog. This was incised to ensure complete release of the median nerve. The median nerve was found to be edematous, intact, and free of compression after the release. The incision was then closed, and the patient was placed in a soft dressing for 2 weeks, with early activity advancement. At 1 year of follow-up, the patient reported improvement in wrist pain and decreased numbness in her hand. Written informed consent was provided by the patient for the publication of her case and the associated images.

## Discussion

Ulnar dysmelia is a rare congenital condition. It is also called ulnar “hemimelia” and is characterized by the partial or complete longitudinal absence of the ulnar bone.^3^ It is a postaxial developmental defect, and its presentation is heterogeneous, ranging from isolated ulnar deficiency to affecting the elbow, hand, and even lower extremities.^4^ The extent of ulnar deficiency dictates the morphology of the elbow and, subsequently, the degree of motion. Patients with the ulna truncated distally have relatively preserved function, with minimal effect on the range of motion.^5^ In contrast, in the total absence of the ulna, the radius develops abnormally to articulate with the humerus. On this more severe end of the spectrum, either the radius is dislocated and replaces the ulna to form a pseudoarticulation with some preserved range of motion or the elbow presents as a radiohumeral synostosis fixed in either flexion or extension.^5^^,^^6^ Our patient’s elbow lacked the capitellum, and her elbow consisted of a pseudoarticulation of the proximal radius with the humerus, which allowed a limited range of motion. Patients with similar elbow morphology have been reported to have more functional limbs than those with a humeroradial synostosis.^4^^,^^6^

Similarly, the wrist and hand have a wide range of deformities, with the presence of a variable number of carpal bones and finger rays.^6^ It is thought that the distal carpals and rays are directed to develop via a signal from the preceding bone in the forearm in a proximodistal fashion. As such, the presence of the ulna directs the development of the ulnar carpals, metacarpals, and rays; similarly the presence of a radius directs the development of radial bones. A case series by Harrison et al^7^ supported this hypothesis with 3 cases of ulnar dimelia with varying numbers (6, 7, and 8) of ulnar-like fingers and no digit that appeared like a thumb. They concluded that the ulna dictates the presence of the capitate, hamate, triquetral, and 3 metacarpals around the central lunate, trapezoid, and middle metacarpal because each patient had duplications of these bones.^7^ Many of the reported cases of ulnar hemimelia had fewer than the usual 5 digits, and the absence of bones was noted on the ulnar side of the hand. The most common presentation reported is a 3-fingered hand, which our patient had on the left side.^3^^,^^6^ Walker et al^4^ reported that more severe cases of ulnar dysmelia also had more severe elbow and finger abnormalities, theorized to be a dose-dependent effect. The same study also found that patients with fewer associated lower extremity defects had less severe cases of ulnar dysmelia.

Despite the absence of normal anatomy, both the physical examination and diagnostic studies performed in our patient were consistent with CTS. She had a positive Phalen test result, which has a reported specificity of 74%, and confirmatory EMG findings.^8^ Interestingly, this patient developed symptoms of CTS at the relatively young age of 23 years, whereas the average age of patients diagnosed with CTS was 52 years in the Swedish population study.^1^ The patient notably had a flexion contracture of the left wrist, which was unaffected by orthosis fabrication. It is possible that her unusual anatomy restricted the volume of the carpal tunnel more than in a normal wrist, which caused her symptoms to develop earlier in life. Other authors have similarly reported cases of CTS developing in patients with congenital abnormalities, including radial deficiency and scaphoid hypoplasia.^9^^,^^10^ These patients also had abnormal carpal tunnel anatomy but still developed CTS and were diagnosed at an unusually young age.^10^ It is worth noting that our patient had dysmorphic anatomy bilaterally but was only diagnosed with CTS on the left wrist. Her right wrist had a greater range of motion and a less severe contracture, which may explain her clinical presentation.

This case adds to the limited literature on ulnar dysmelia. Although we are unable to comment on whether our patient’s anatomy caused CTS, her case serves as a reminder that CTS can still occur in patients who lack anatomic components of the carpal tunnel.
